# New daily persistent headache with May-Thurner physiology and spinal epidural venous congestion: treatment with ascending lumbar vein embolization

**DOI:** 10.1093/bjrcr/uaaf045

**Published:** 2025-09-09

**Authors:** Samuel B Ogunlade, Todd D Rozen, Andrew R Lewis, Beau B Toskich, Zlatko Devcic

**Affiliations:** Division of Interventional Radiology, Department of Radiology, Mayo Clinic, Jacksonville, FL 32224, United States; Department of Neurology, Mayo Clinic, Jacksonville, FL 32224, United States; Division of Interventional Radiology, Department of Radiology, Mayo Clinic, Jacksonville, FL 32224, United States; Division of Interventional Radiology, Department of Radiology, Mayo Clinic, Jacksonville, FL 32224, United States; Division of Interventional Radiology, Department of Radiology, Mayo Clinic, Jacksonville, FL 32224, United States

**Keywords:** spinal epidural venous plexus, May-Thurner, interventional radiology, headache, ascending lumbar vein embolization

## Abstract

May-Thurner physiology (MTP) can lead to various congestion syndromes due to compression of the left common iliac vein (LCIV) by the right common iliac artery (RCIA). This compression may result in venous reflux through the lumbar vein, leading to congestion of the spinal epidural venous plexus (EVP), which could contribute to refractory headaches. This case report details the clinical course of a patient with severe refractory new daily persistent headache associated with MTP who underwent ascending lumbar vein (ALV) embolization. The patient is a 59-year-old female with a 3-year history of daily persistent headache which failed multiple migraine prevention therapies and minimally invasive procedures. Imaging studies revealed significant LCIV compression by the RCIA, retrograde ALV flow, and EVP congestion. The patient underwent ALV embolization, resulting in significant symptomatic relief. At the 22-month follow-up, headache severity reduced by 80%, with the patient no longer requiring routine headache medications. Additionally, treatment of associated pelvic congestion syndrome through gonadal vein embolization resolved longstanding associated pelvic pain and pressure. This case highlights the role of venous congestion in refractory headache syndromes and underscores the potential of targeted venous interventions, such as embolization, in their management. The findings expand on emerging evidence linking venous compression syndromes to headache pathophysiology and support exploring interventional strategies as viable treatment options for selected patients. Further research is needed to validate these findings and establish evidence-based guidelines for clinical practice.

## Introduction

New daily persistent headache (NDPH) is a primary headache disorder marked by a daily headache from onset persisting for at least 3 months in duration.^1^ NDPH is noted to be anything but benign, as it is recognized as one of the more treatment-refractory headache syndromes, and it severely impairs quality of life.^1^ Our recent findings connecting pathologies outside of the central nervous system, including nutcracker physiology (NCP) with spinal epidural venous congestion, have opened the door to new effective treatment strategies.^2–4^

May-Thurner syndrome (MTS) occurs when the left common iliac vein (LCIV) is compressed by the right common iliac artery (RCIA), causing venous obstruction, elevated pressures, and pelvic or lower extremity symptoms like pain, varicosities, and swelling.^5^^,^^6^ When, however, there are no associated pelvic or lower extremity symptoms, it is referred to as May-Thurner physiology (MTP). MTS has been linked to vascular complications, including deep vein thrombosis,^5^ pulmonary embolism,^7^ and pelvic congestion syndrome (PCS).^6^ Studies highlight connections between elevated venous pressure in conditions such as NCP and increased intracranial pressure contributing to headache syndromes.^2–4^^,^^8^ NCP involves narrowing of the left renal vein (LRV) between the superior mesenteric artery and aorta, causing retrograde lumbar vein (RLV) flow and epidural venous plexus (EVP) congestion.^3^ Recent studies^2^^,^^4^ show that lumbar vein embolization may reduce EVP congestion, normalize cerebrospinal fluid (CSF) dynamics, and resolve refractory headaches, supporting this therapeutic approach. Similarly, ascending lumbar vein (ALV) embolization offers a novel approach to addressing RLV flow and EVP congestion from MTP, potentially alleviating elevated intracranial pressure and reducing headaches. As of yet, MTP has not been linked to daily persistent headaches.

This case report presents the clinical course of a patient diagnosed with NDPH associated with MTP who underwent ALV embolization as a therapeutic intervention. By highlighting the therapeutic benefits and procedural outcomes of this innovative approach, this case report aims to advance the understanding of the vascular contributions to NDPH and stimulate further investigation into interventional strategies for its management.

## Case presentation

A 59-year-old female was initially evaluated and referred by a fellowship-trained headache neurologist with a history of severe refractory daily headaches. The patient reported a sudden onset of daily headaches approximately 3 years before presentation, with no prior history of headaches. The headache was said to have started as a low-grade, throbbing pain and was located primarily in the occipital region and radiating to the left ear. As her symptoms evolved, the headache worsened, spreading to the frontal area and accompanied by nausea, vomiting, photophobia, phonophobia, lightheadedness, vertigo, and near-syncope. Her headache pain intensity at presentation was rated at a visual analogue scale (VAS) of 6-8/10 and was aggravated by lying on her left side and being placed in the Trendelenburg position. Her worsening in the head down tilt and some relief with CSF volume removal suggested the possibility of an abnormal reset of her CSF pressure to an elevated state. Her outside diagnosis was chronic headache.

The patient’s headaches were refractory to a range of migraine preventive treatments, including gabapentin, propranolol, topiramate, and venlafaxine, as well as minimally invasive procedures, including onabotulinum toxin A injections and greater occipital nerve blocks. Neuroimaging including magnetic resonance imaging (MRI) brain (Supplementary S1), MR venogram (Supplementary S2), CT angiogram of the head (Supplementary S3), and neck (Supplementary S4) did not find a secondary cause including mass lesion, Chiari malformation, cerebral vein thrombosis or sinus stenosis and no aneurysm or dissection. The patient also underwent a lumbar puncture before her initial consultation. She had a normal opening pressure of 14 cmH_2_O, her headache felt somewhat better but only for 1-2 days, after which there was a recurrence back to baseline.

She was prescribed a trial of sustained-release indomethacin at 75 mg once daily, but this was discontinued due to a severe headache exacerbation after only 2 doses. A subsequent trial with acetazolamide (125 mg at bedtime) provided temporary relief, reducing her headache to an average VAS score of 4. However, this improvement was short-lived, and her headaches intensified again after 3 months, even when her acetazolamide dose was increased to 500 mg extended-release twice daily.

**Figure 1.**
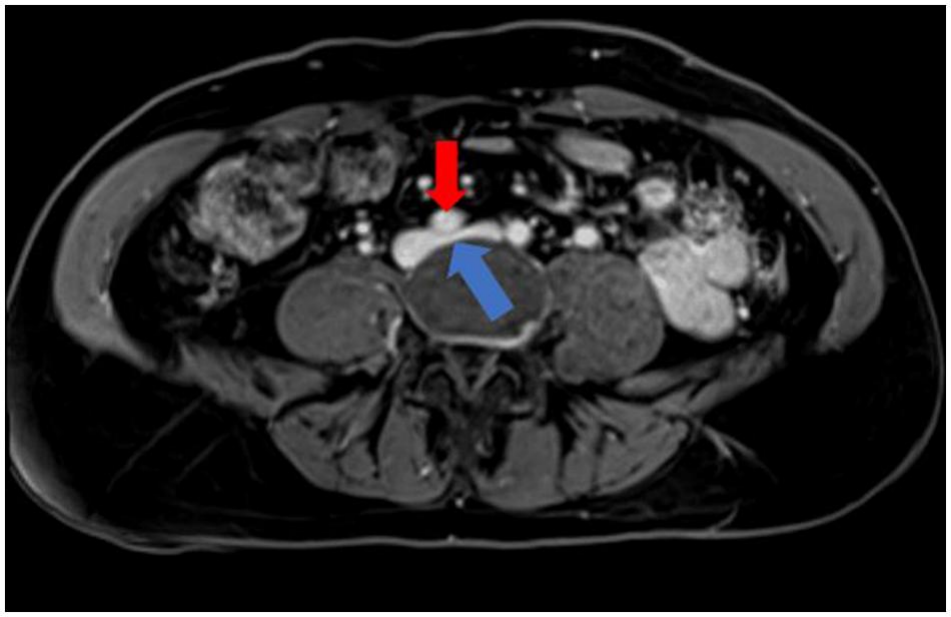
Axial cross-sectional magnetic resonance image demonstrating severe compression of the left common iliac vein (LCIV) (blue arrow) between the right common iliac artery (RCIA) (red arrow) anteriorly and the spine posteriorly. The anteroposterior diameter of the LCIV is narrowed by more than 50% at the compression point when compared with the adjacent venous segment.

Due to the complexity of her refractory headache and the authors’ interest in a possible link to venous congestion syndromes, further imaging was conducted to investigate her venous anatomy. Patient consent was obtained, and the case report complied with the Health Insurance Portability and Accountability Act (HIPAA) regulations. MRI revealed compression of LCIV by the overlying RCIA, consistent with MTP (Figure 1), and time-resolved MR angiography (trMRA) revealed retrograde drainage of a posteriorly based ALV into the EVP, with potential congestion at this level. Although the exact source of EVP congestion was uncertain, it was hypothesized that severe compression of the LCIV by the overlying RCIA may be resulting in retrograde ALV flow and subsequent EVP congestion, contributing to her chronic headaches.

**Figure 2.**
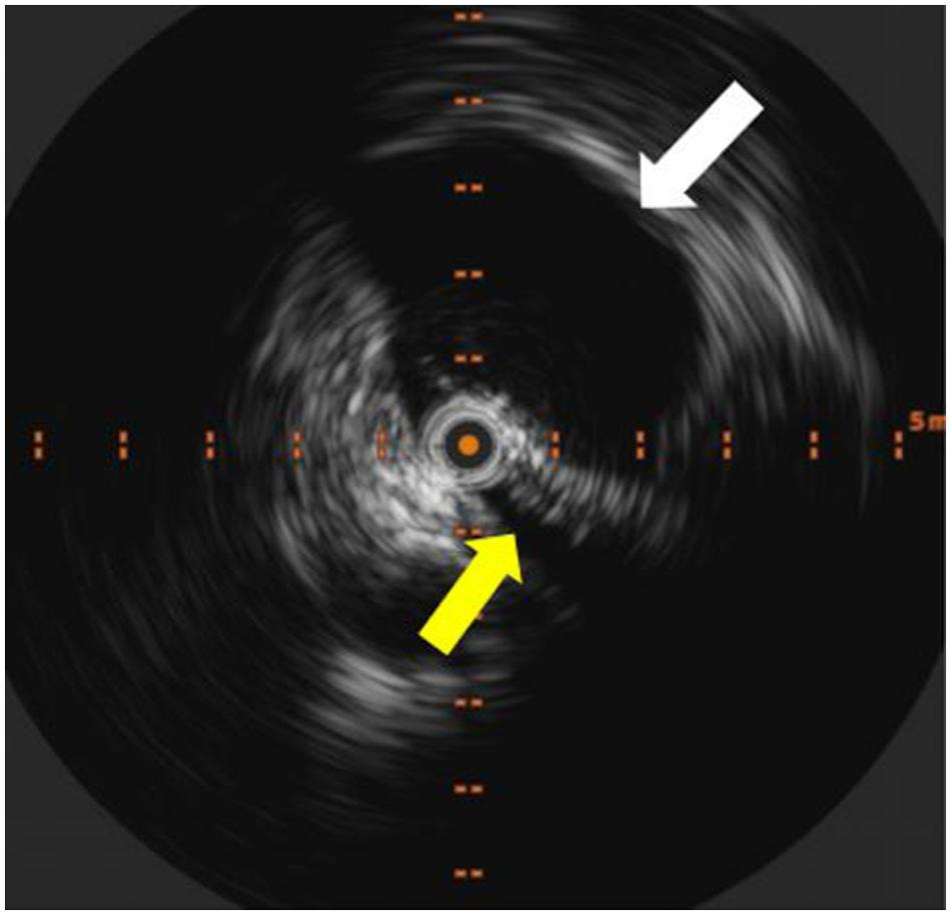
Intravascular ultrasound (IVUS) demonstrates severe LCIV compression (yellow arrow) by the overlying RCIA (white arrow). Abbreviation: LCIV = left common iliac vein; RCIA = right common iliac artery.

**Figure 3.**
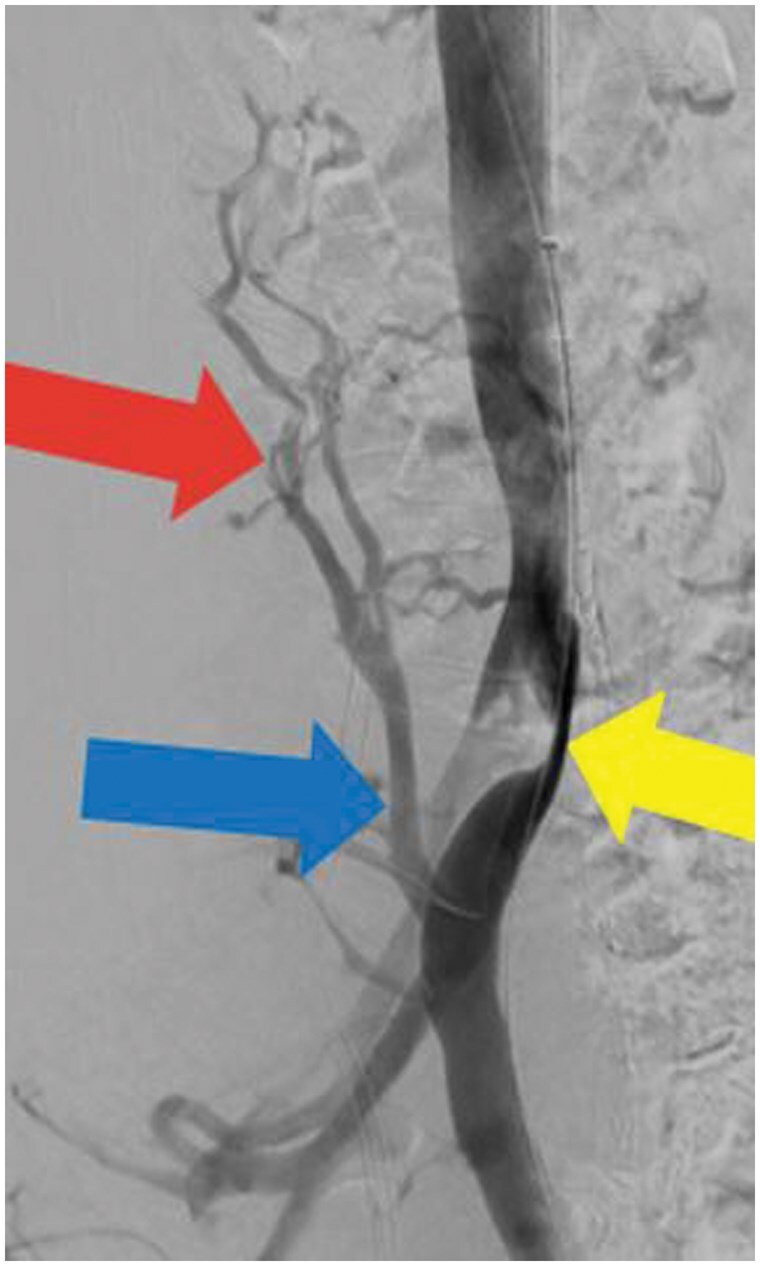
Lateral venography showing severe LCIV compression (yellow arrow) and retrograde ascending lumbar vein flow (blue arrow) with robust epidural venous plexus congestion (red arrow). There is a paucity of contrast in the LCIV subjacent at the site of maximal compression. This was a power injection obtained in the supine position with a pigtail catheter in the left external iliac vein at 6 mL/second for a total volume of 18 mL. Imaging was performed in the lateral projection. Abbreviation: LCIV = left common iliac vein.

**Figure 4.**
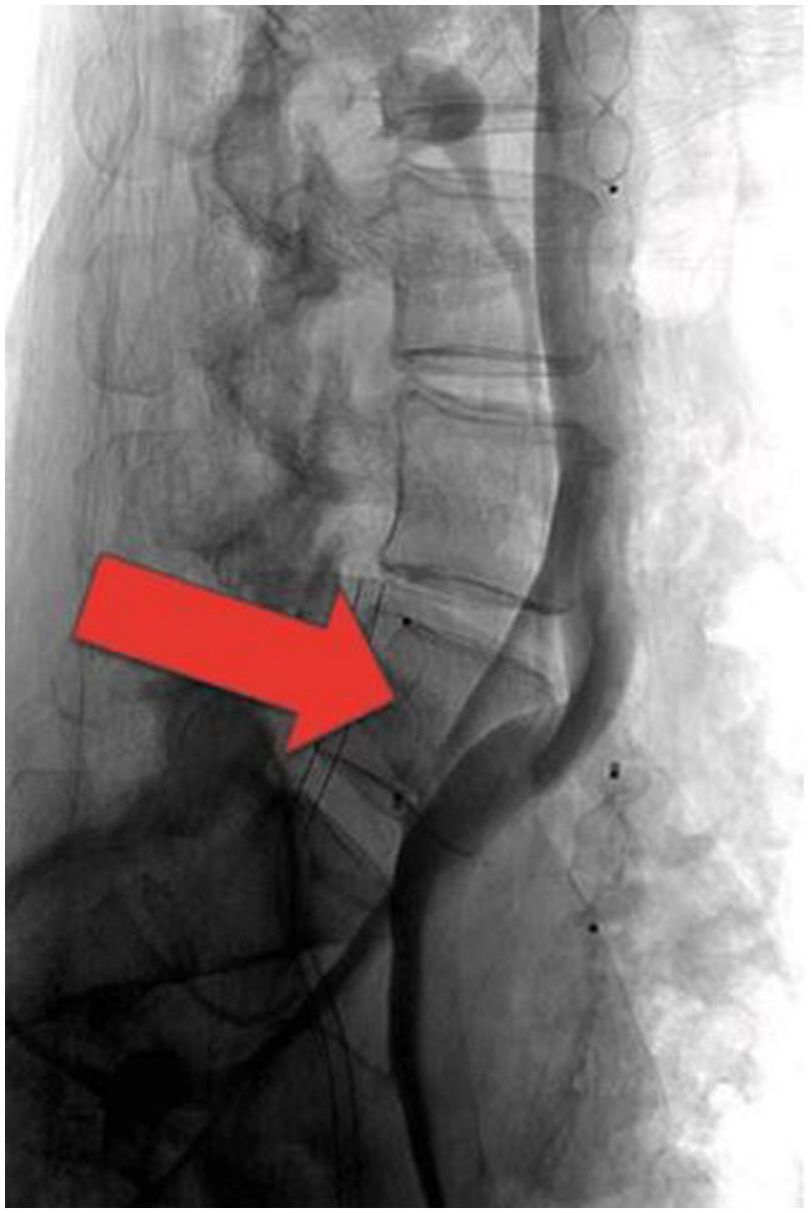
Ascending lumbar vein embolization with plug insertion. This was a power injection obtained in the supine position with a pigtail catheter in the left external iliac vein at 6 mL/second for a total volume of 18 mL. Imaging was performed in the lateral projection.

**Figure 5.**
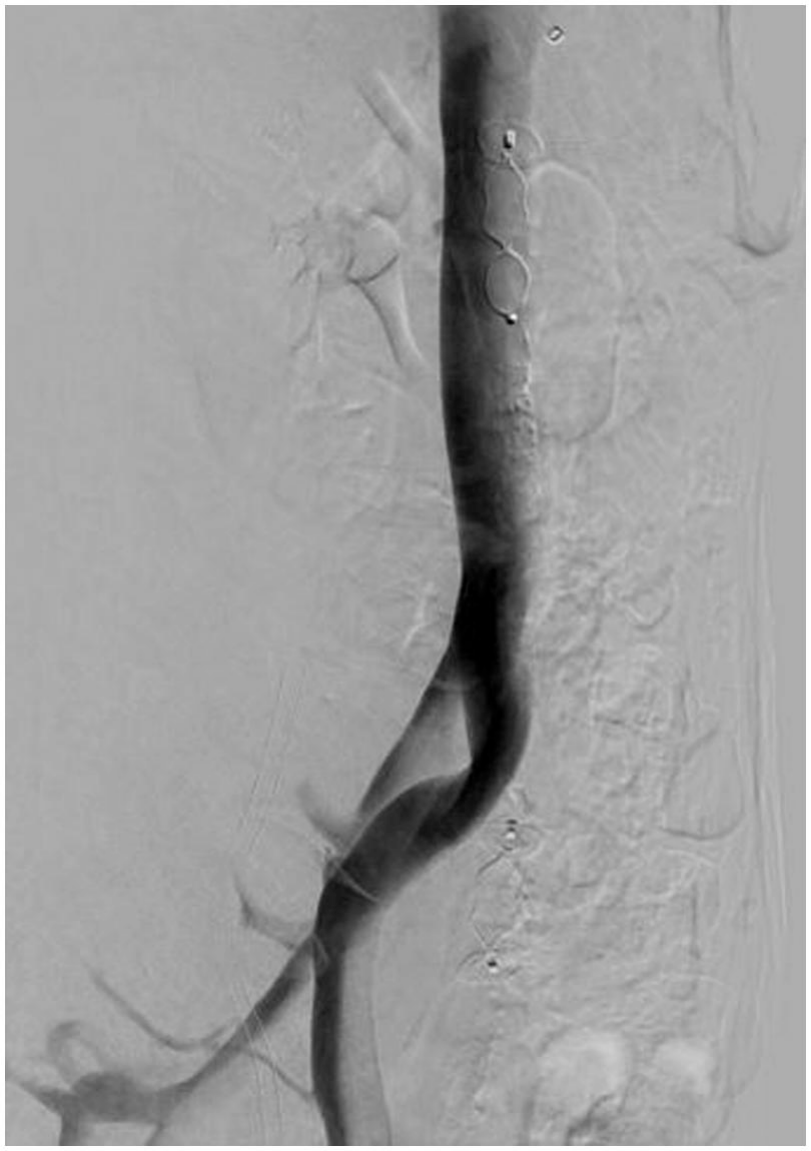
Post-embolization image showing no flow into the ascending lumbar vein and no EVP congestion. This was a power injection obtained in the supine position with a pigtail catheter in the left external iliac vein at 6 mL/second for a total volume of 18 mL. Imaging was performed in the lateral projection. Abbreviation: EVP = epidural venous plexus.

Catheter-based venography and intravascular ultrasound (IVUS) were subsequently performed to assess venous haemodynamics in more detail. A pressure gradient of 2 mmHg was measured across the stenotic segment of the LCIV to the inferior vena cava (IVC). IVUS demonstrated severe narrowing of the LCIV (Figure 2), and venography confirmed retrograde flow through the ALV due to severe LCIV compression (Figure 3). To relieve the EVP congestion hypothesized to be contributing to the patient’s headache, plug embolization of the ALV was performed (Figure 4). The objective of this intervention was to stop the abnormal retrograde venous reflux into the EVP (Figure 5), thus reducing the EVP congestion that may be aggravating her headaches.

Following ALV embolization, the patient experienced significant improvement in her headache symptoms. At her 22-month follow-up, she reported an 80% reduction in headache severity. Her headaches, previously rated between VAS score 6 and 8, were now 0 on most days, with occasional flare-ups reaching only 3/10 about once per week. She now manages these minor episodes with acetazolamide as needed and no longer requires her previous schedule of headache medications.

After the ALV was embolized to relieve the patient’s headaches, attention was turned to her longstanding symptoms of PCS. These included significant pelvic pain and discomfort, which had been present for many years. trMRA demonstrated retrograde flow through the left gonadal vein, caused by compression of the LRV. This finding explained the source of the pelvic venous congestion (Supplementary S5). To ensure that embolization of the gonadal vein would be safe, a temporary balloon occlusion test was done using a Fogarty balloon. This confirmed no significant rise in pressure from the LRV to the IVC, confirming that embolization could be safely performed. The patient then underwent left gonadal vein and pelvic variceal embolization using plugs and sodium tetradecyl sulphate (Supplementary S6), which directly addressed the source of her PCS-related symptoms.

At her 22-month follow-up, the patient reported that she no longer experienced pelvic pain or pelvic discomfort. Follow-up imaging also showed no further evidence of pelvic venous congestion (Supplementary S7).

## Discussion

There is increasing evidence that venous compression syndromes can contribute to intracranial and spinal venous congestion, disrupting CSF dynamics and precipitating refractory headache syndromes.^2–4^^,^^8^ The treatment approach, in this case, was informed by previous insights gained from managing NCP, where the compression of the LRV causes a retrograde venous flow through the second lumbar vein (L2LV) and into the EVP.^2^^,^^4^ In NCP patients, elevated EVP pressure has been shown to contribute to intracranial pressure fluctuations, manifesting as refractory headaches.^2^^,^^3^ Embolizing the L2LV in these patients has effectively reduced EVP congestion and yielded significant headache relief, as documented in Devcic et al.^2^ and the case series by Rozen et al.^4^ These findings prompted the exploration of similar venous mechanisms in other compression syndromes, including MTP, to alleviate neurological symptoms, such as this case, which otherwise would have been attributed solely to idiopathic intracranial hypertension or other primary headache disorders.

In MTP, compression of the LCIV by the RCIA results in venous stasis and increased venous pressure within the left iliac system, often resulting in collateral drainage through the ALV into the EVP. The EVP, a critical drainage pathway for spinal and intracranial venous blood, can become engorged when subjected to excessive venous flow, potentially leading to increased intracranial pressure and chronic headaches. The hypothesis that EVP congestion can influence intracranial CSF dynamics and lead to headache^3^^,^^8^ is supported by the patient’s positional symptom exacerbation, with increased headache intensity in positions that raise venous and CSF pressure (eg, Trendelenburg position), suggesting impaired venous drainage may be playing a role in symptomatology.

Given these pathophysiological similarities between MTP and NCP, we hypothesized that ALV embolization could similarly interrupt abnormal venous drainage pathways and alleviate pressure within the spinal venous system. Diagnostic imaging, including MRI and IVUS, showed significant LCIV compression, and venography confirmed the retrograde flow through the ALV, and congestion in the epidural venous systems.

Plug embolization was performed to occlude the ALV and reduce abnormal venous drainage into the spinal venous system. Although iliac vein stenting is widely accepted as first-line treatment for MTS in patients with symptomatic lower extremity venous obstruction or claudication, it was not used in this case, though initially considered. This decision followed a careful evaluation of the patient’s clinical presentation and symptom pathophysiology. The patient had no leg swelling or pain typically linked to iliac vein compression. Instead, her main complaint was NDPH, hypothesized to result from intracranial venous congestion due to retrograde ALV flow into the EVP. While stenting can relieve iliac vein compression, it does not directly address abnormal collaterals. In this patient, symptoms were driven by collateral drainage through the ALV into the EVP, causing intracranial venous hypertension. Embolizing the ALV offered a more targeted, physiologically appropriate solution by directly eliminating the reflux pathway responsible for her headaches.^2^^,^^4^

Embolization of the ALV was considered a less invasive alternative, and based on clinical judgement, it was hypothesized to carry a lower risk of complications compared to stenting in this case. While stenting is commonly used for MTS, it carries risks such as stent migration^9^ and in-stent thrombosis or occlusion.^10^ As the patient had no lower extremity symptoms, stenting was deemed potentially unnecessary, with its risks possibly outweighing benefits. This decision was informed by multidisciplinary evaluation and the patient’s preferences, prioritizing treatment of her most disabling symptom—headache—while avoiding more invasive iliac vein procedures.

Although MTP can progress to clinically significant MTS with lower extremity symptoms or thrombosis, this patient showed no such signs. As a precaution, close clinical monitoring and follow-up imaging have been implemented to detect any sign of progression. Early identification of hemodynamic changes will allow timely intervention if MTS-related symptoms develop.

It is important to acknowledge that leaving the underlying MTP untreated may lead to new collateral pathway development over time. In this case, the patient’s persistent headache was successfully resolved with targeted embolization of symptomatic collaterals, and she exhibited no symptoms typically associated with iliac vein compression, such as leg swelling or deep vein thrombosis. As a result, no additional intervention for MTP was planned at the time of this report. Cross-pelvic collateral veins were intentionally preserved during the procedure to maintain sufficient venous drainage and reduce the risk of worsening outflow obstruction. The patient remains under clinical and imaging follow-up to monitor for symptom recurrence or new collateral formation. At the 22-month follow-up, she remained asymptomatic with no evidence of recurrence.

This intervention provided both symptomatic and functional relief as the 22-month follow-up demonstrated the patient’s headache frequency and intensity had decreased significantly, with her VAS score dropping from 6-8/10 to 0-3/10. This substantial improvement allowed her to discontinue scheduled headache medications and rely only on occasional, as-needed doses of acetazolamide. Additionally, her pelvic congestion symptoms, which had been severe and unresponsive to conservative treatments, completely resolved post-procedure.

The patient’s notable response expands on the emerging evidence of venous compression syndromes as contributors to refractory headache disorders. It also raises the possibility that by identifying and targeting venous collaterals that act as decompressive pathways into the spinal or intracranial compartments, interventional procedures like percutaneous ALV embolization may offer viable, minimally invasive treatment options for patients with vascular headache aetiologies unresponsive to conventional therapies.

## Conclusion

This case highlights the importance of considering venous compression disorders, including MTP, in the differential diagnosis of refractory headache conditions. It underscores the potential for targeted outpatient vascular interventions, such as ALV embolization, to relieve symptoms by addressing the extracranial venous abnormalities contributing to abnormal EVP and CSF pressure dynamics. Further research, including prospective evaluations and long-term outcome tracking, is necessary before routine ALV embolization can be recommended due to concerns about the potential placebo effect. Nonetheless, this case contributes to the expanding literature supporting venous interventions as promising therapeutic options for carefully selected patients with complex headache syndromes. These patients must be treated in a multidisciplinary effort with a neurologist in the work-up and close follow-up.

## Learning points

- New daily persistent headache may have underlying extracranial vascular causes, such as May-Thurner physiology (MTP), that are not apparent on standard neuroimaging.
- Spinal epidural venous congestion, linked to pelvic venous outflow obstruction, can alter cerebrospinal fluid dynamics and contribute to treatment-refractory headache syndromes.
- Ascending lumbar vein embolization can alleviate EVP congestion and represents a promising therapeutic option for headaches associated with MTP.
- This case supports expanding the diagnostic workup of refractory headaches to include pelvic and spinal venous assessment when central nervous system findings are negative.
- Recognition of venous outflow abnormalities as a contributor to intracranial pressure dysregulation opens up new interventional pathways in headache management.

## Supplementary Material

uaaf045_Supplementary_Data
