# Supplementary material for: New daily persistent headache with May-Thurner physiology and spinal epidural venous congestion: treatment with ascending lumbar vein embolization
Source: BJR Case Rep. 2025 Sep 9;11(5):uaaf045. doi: 10.1093/bjrcr/uaaf045 (PMC12449044; doi:10.1093/bjrcr/uaaf045)
Supplement: uaaf045_Supplementary_Data [file uaaf045_supplementary_data.zip › MTP Case report supplementary BJR final.pptx]

## Slide 1
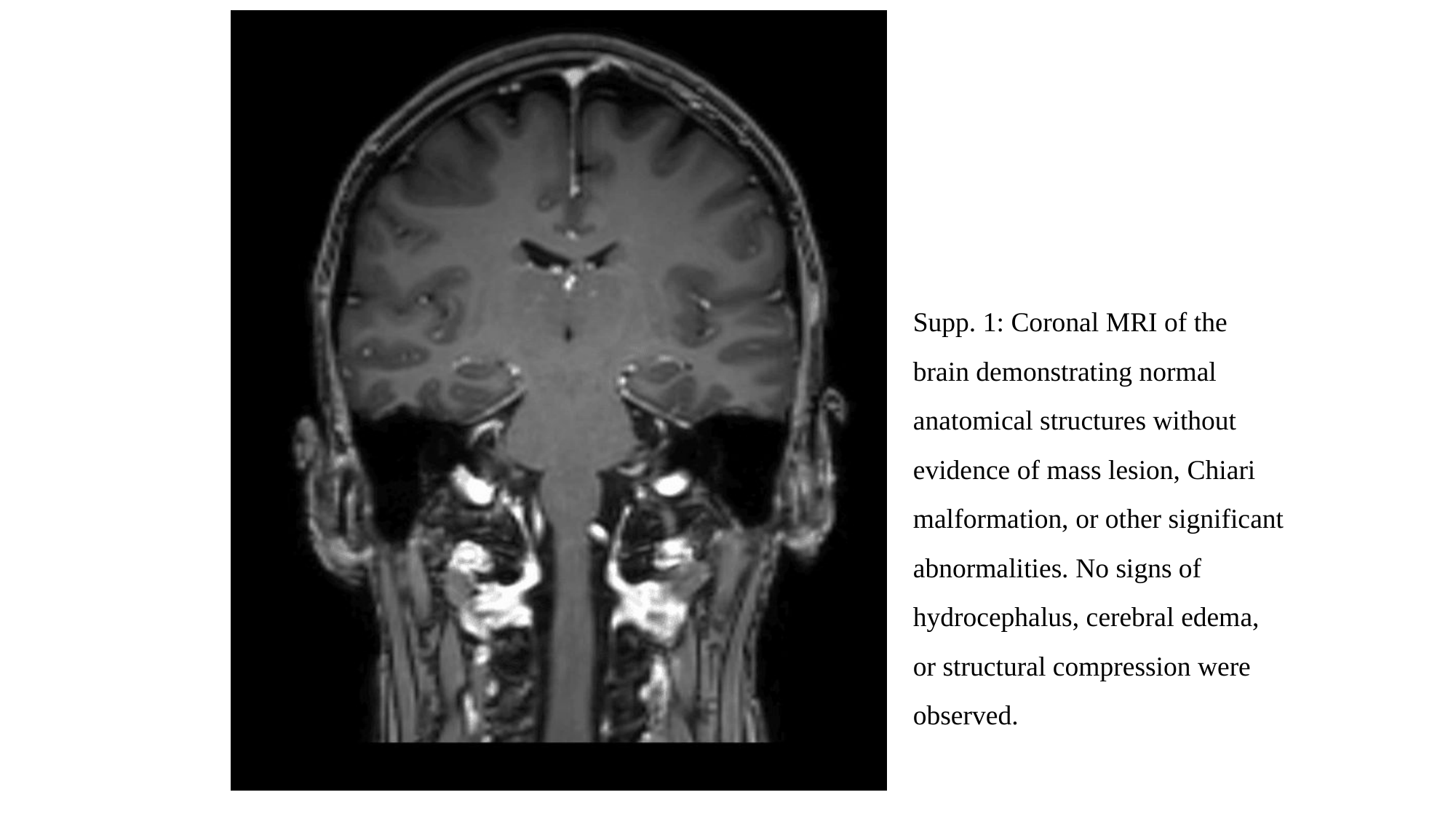

Supp. 1: Coronal MRI of the brain demonstrating normal anatomical structures without evidence of mass lesion, Chiari malformation, or other significant abnormalities. No signs of hydrocephalus, cerebral edema, or structural compression were observed.

## Slide 2
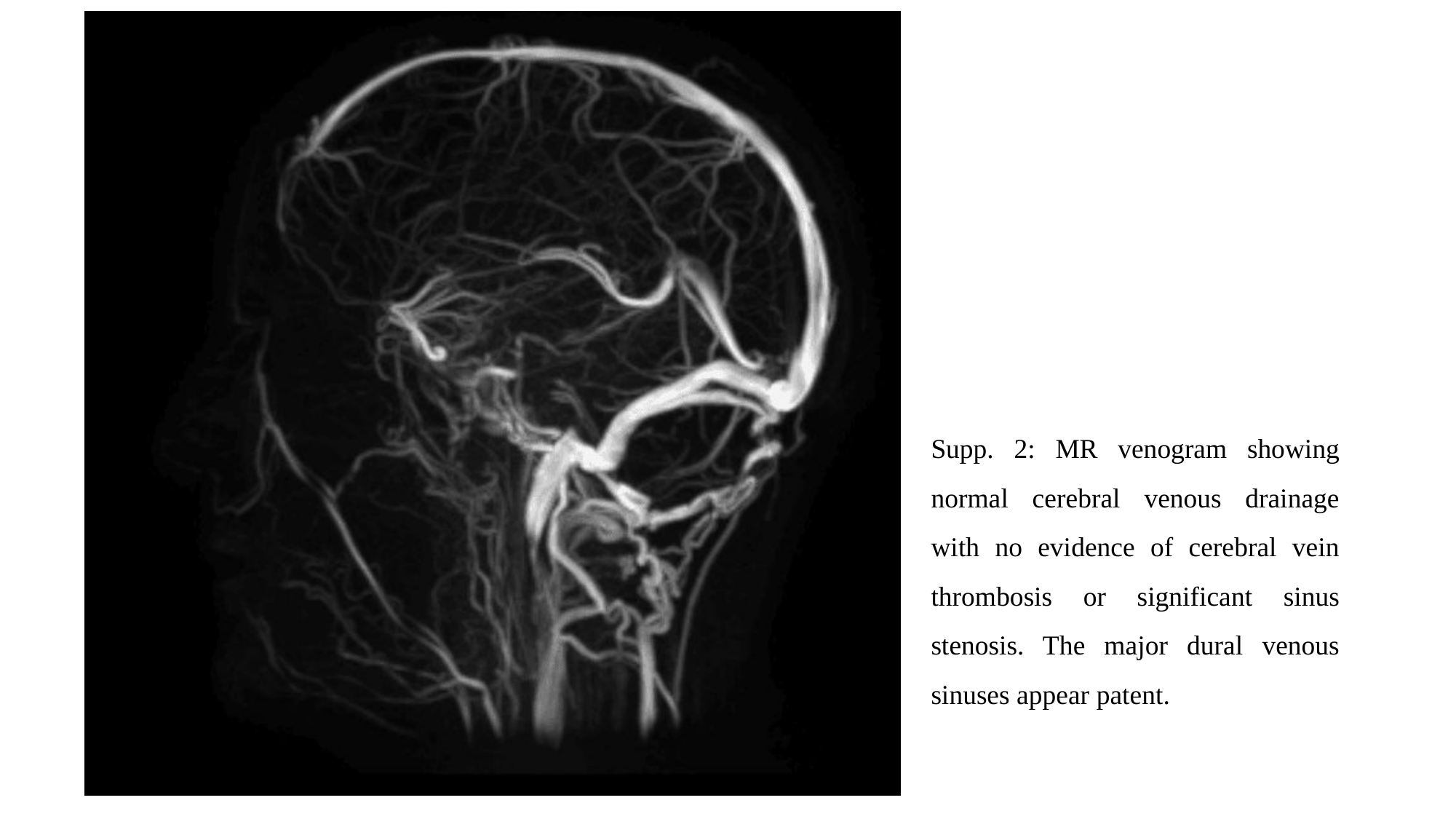

Supp. 2: MR venogram showing normal cerebral venous drainage with no evidence of cerebral vein thrombosis or significant sinus stenosis. The major dural venous sinuses appear patent.

## Slide 3
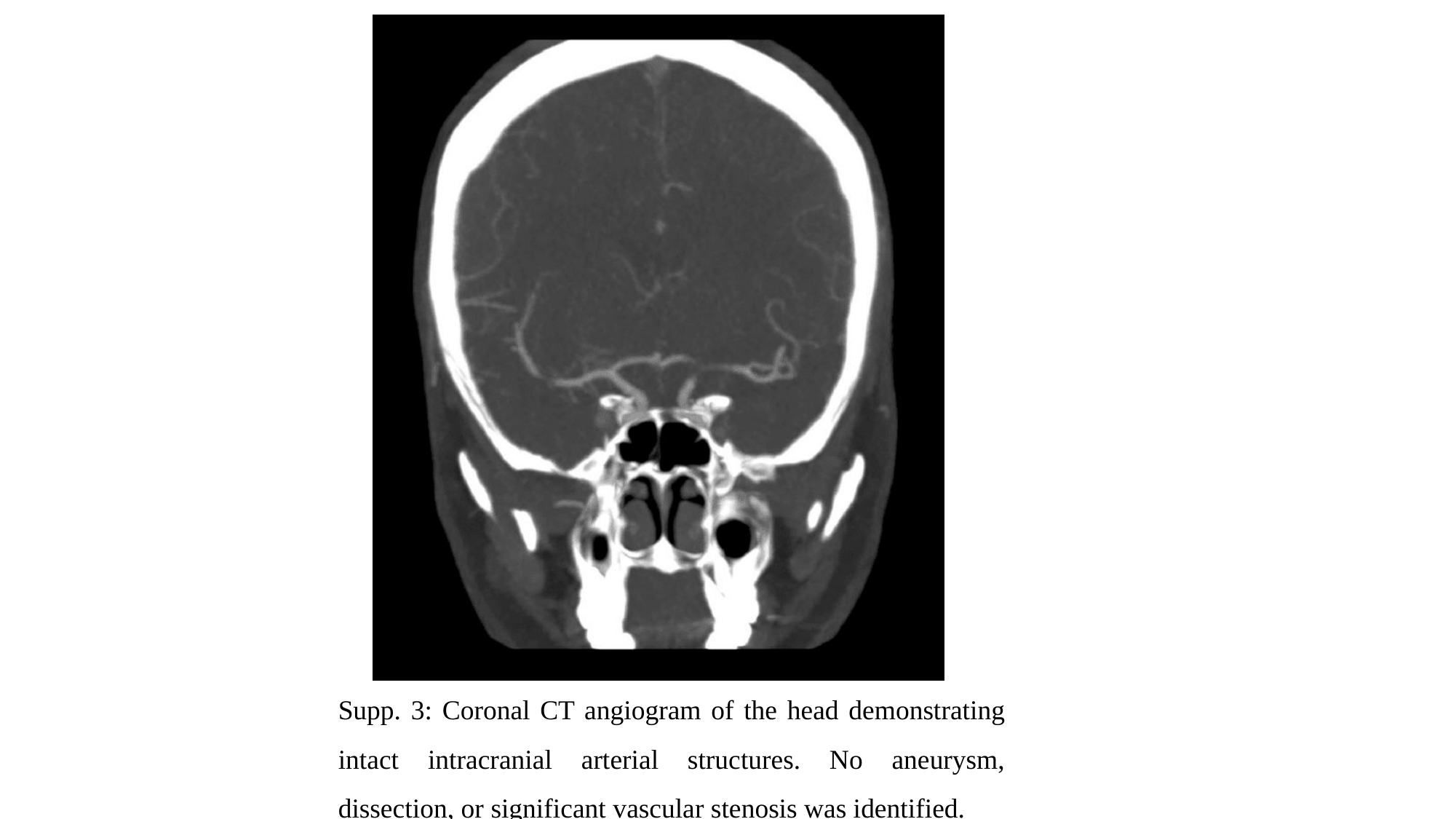

Supp. 3: Coronal CT angiogram of the head demonstrating intact intracranial arterial structures. No aneurysm, dissection, or significant vascular stenosis was identified.

## Slide 4
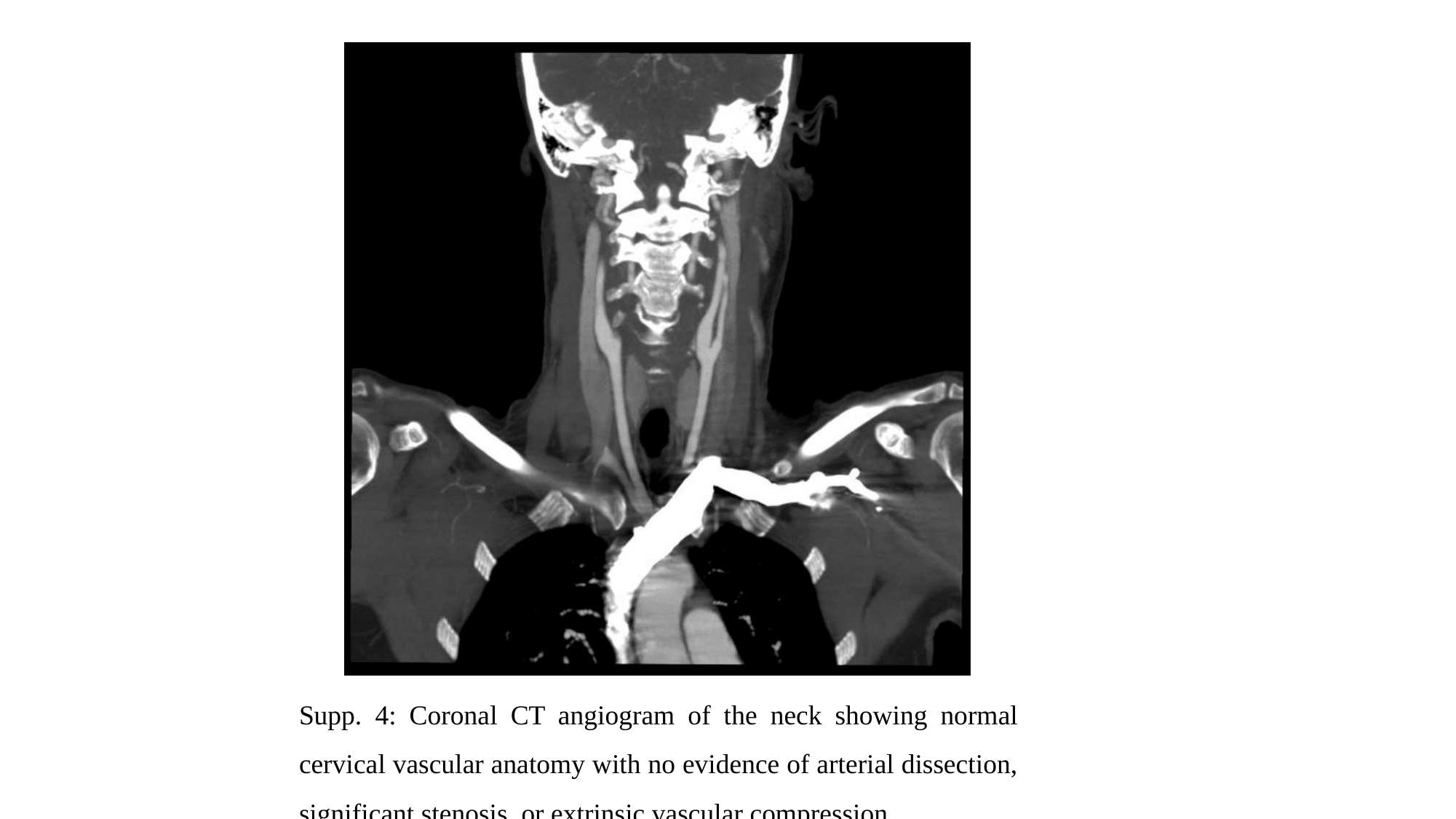

Supp. 4: Coronal CT angiogram of the neck showing normal cervical vascular anatomy with no evidence of arterial dissection, significant stenosis, or extrinsic vascular compression.

## Slide 5
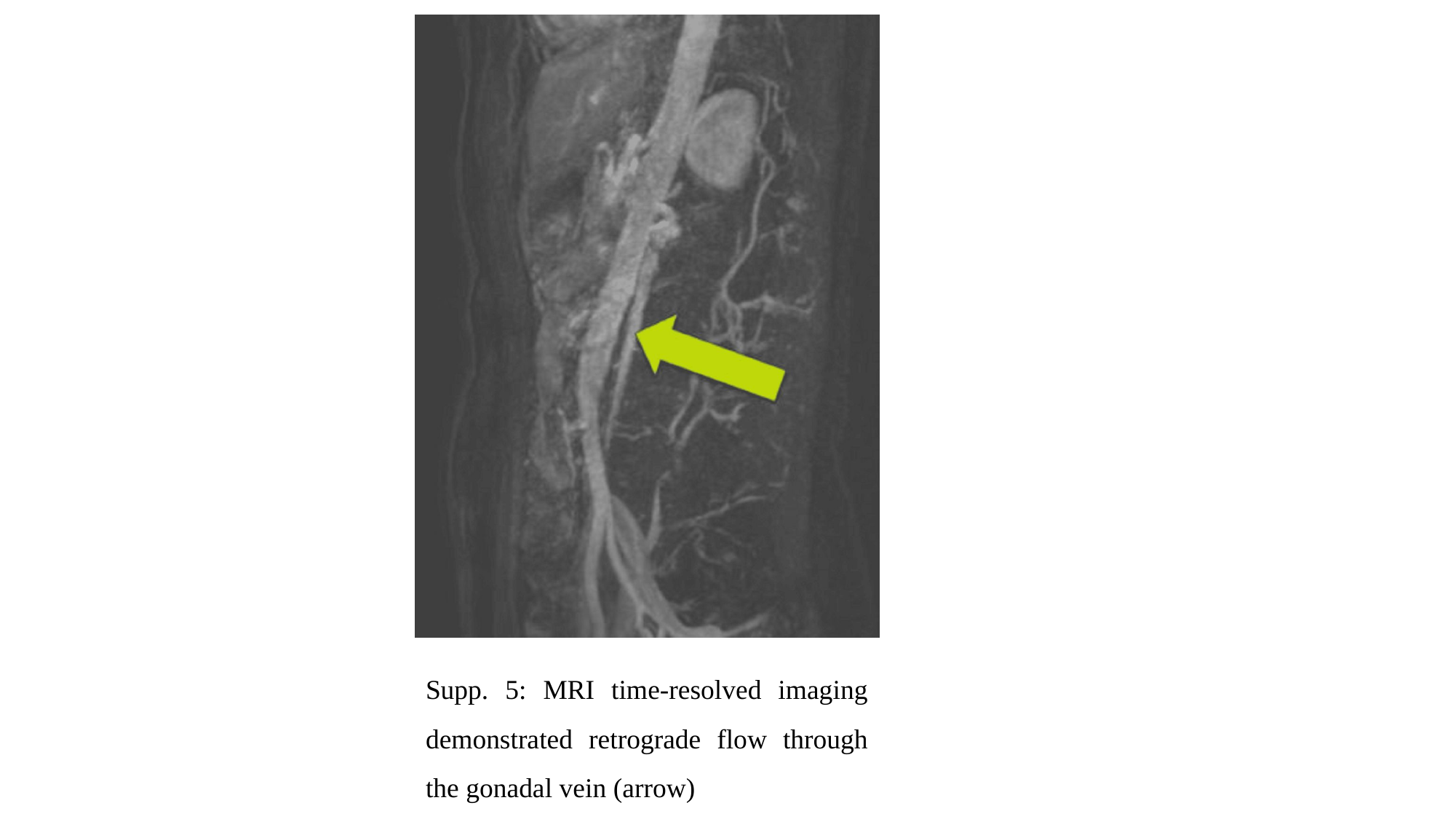

Supp. 5: MRI time-resolved imaging demonstrated retrograde flow through the gonadal vein (arrow)

## Slide 6
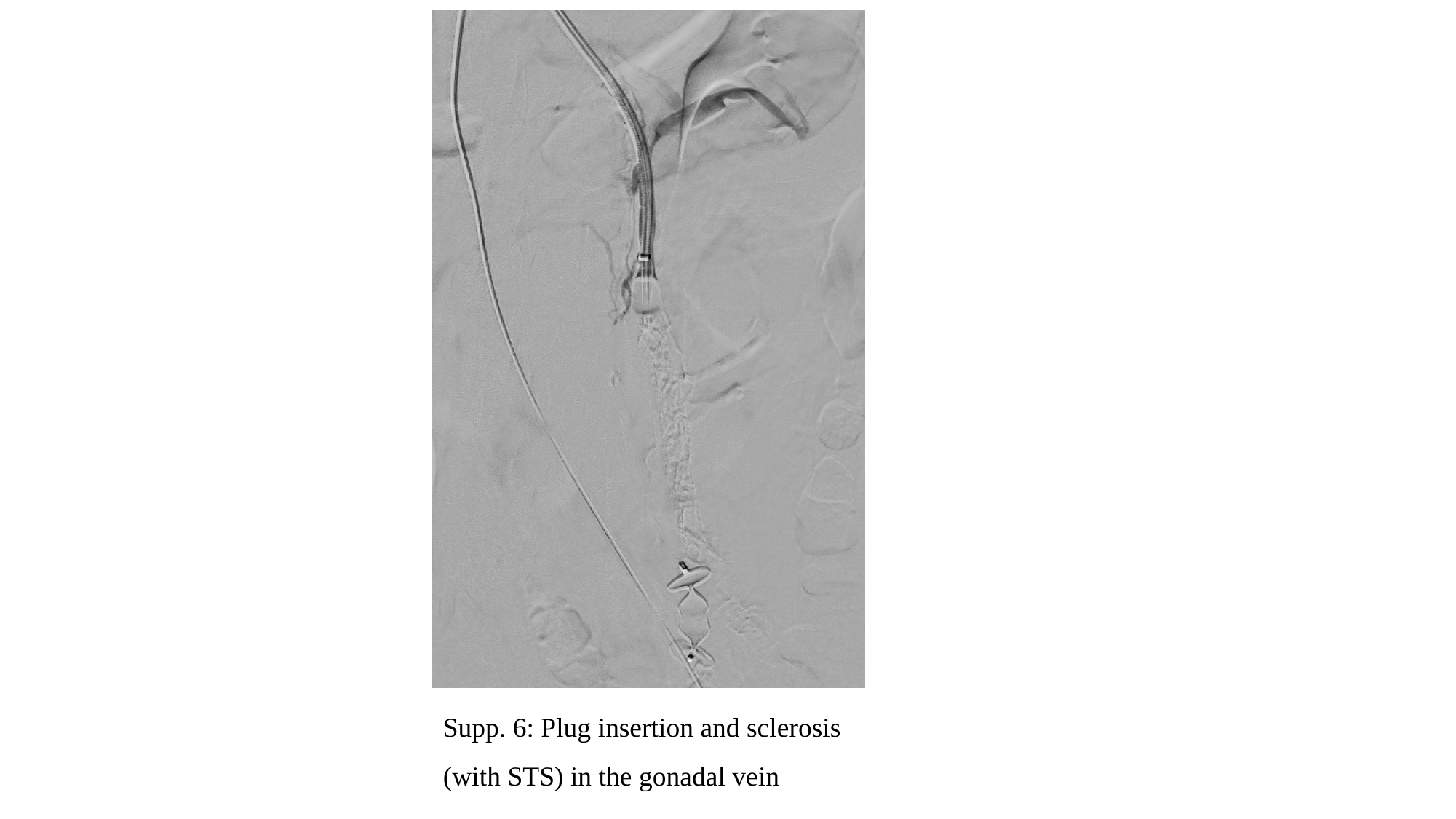

Supp. 6: Plug insertion and sclerosis (with STS) in the gonadal vein

## Slide 7
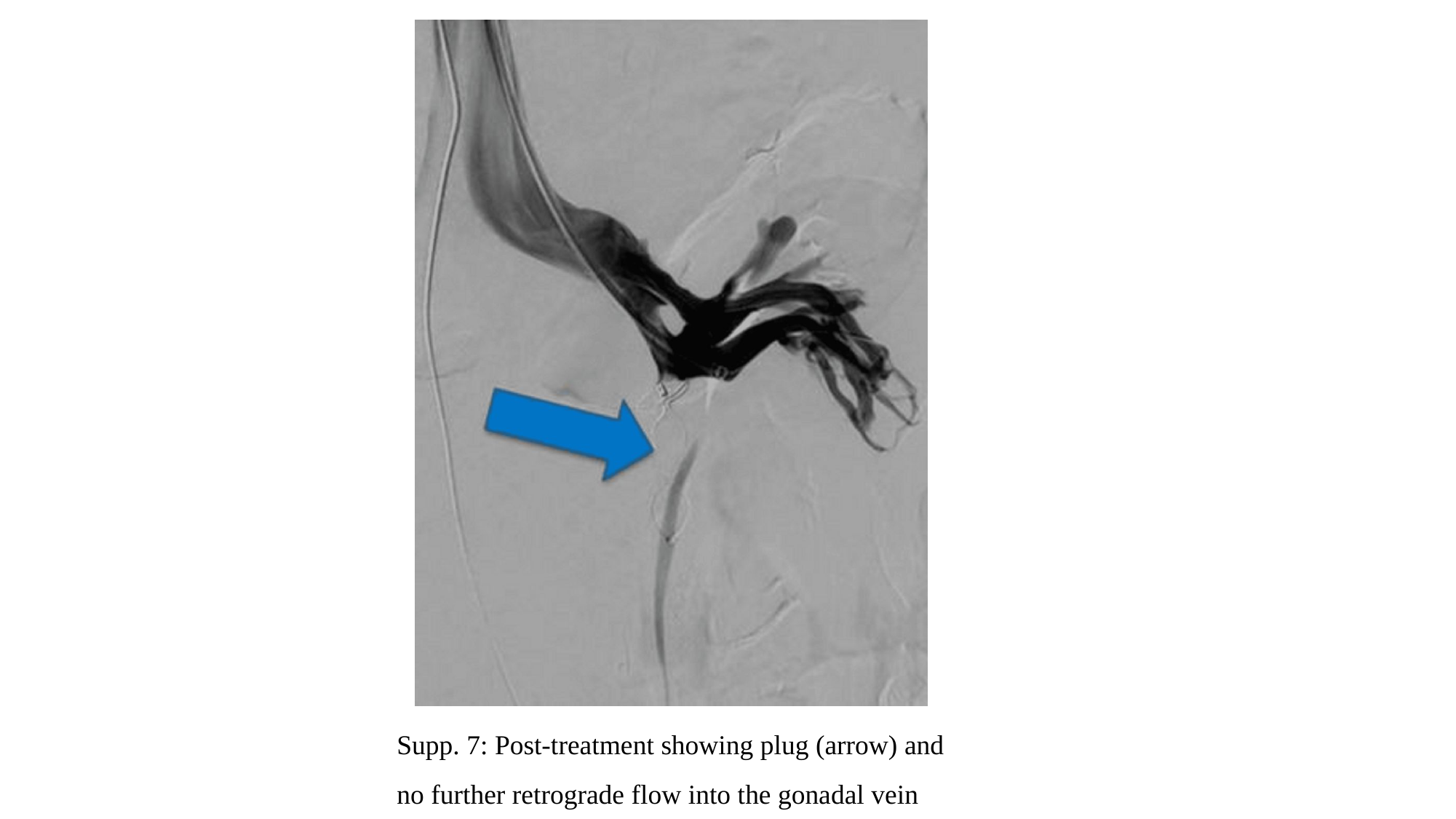

Supp. 7: Post-treatment showing plug (arrow) and no further retrograde flow into the gonadal vein
